# Self-assembled PEGylated albumin nanoparticles (SPAN) as a platform for cancer chemotherapy and imaging

**DOI:** 10.1080/10717544.2018.1489430

**Published:** 2018-07-25

**Authors:** Jung Eun Lee, Myung Goo Kim, Yeon Lim Jang, Min Sang Lee, Nak Won Kim, Yue Yin, Jong Han Lee, Su Yeon Lim, Ji Won Park, Jaeyun Kim, Doo Sung Lee, Sun Hwa Kim, Ji Hoon Jeong

**Affiliations:** a School of Pharmacy, Theranostic Macromolecules Research Center, Sungkyunkwan University, Suwon, Republic of Korea;; b Center for Theragnosis, Biomedical Research Center, Korea Institute of Science and Technology, Seoul, Republic of Korea;; c School of Chemical Engineering, Theranostic Macromolecules Research Center, Sungkyunkwan University, Suwon, Republic of Korea

**Keywords:** Drug dissolution, oil-free formulation, human serum albumin, self-assembled nanoparticle, systemic cancer therapy

## Abstract

Paclitaxel (PTX) is used as a major antitumor agent for the treatment of recurrent and metastatic breast cancer. For the clinical application of PTX, it needs to be dissolved in an oil/detergent-based solvent due to its poor solubility in an aqueous medium. However, the formulation often causes undesirable complications including hypersensitivity reactions and limited tumor distribution, resulting in a lower dose-dependent antitumor effect. Herein, we introduce a facile and oil-free method to prepare albumin-based PTX nanoparticles for efficient systemic cancer therapy using a conjugate of human serum albumin (HSA) and poly(ethyleneglycol) (PEG). PTX were efficiently incorporated in the self-assembled HSA-PEG nanoparticles (HSA-PEG/PTX) using a simple film casting and re-hydration procedure without additional processes such as application of high pressure/shear or chemical crosslinking. The spherical HSA-PEG nanoparticle with a hydrodynamic diameter of ca. 280 nm mediates efficient cellular delivery, leading to comparable or even higher cytotoxicity in various breast cancer cells than that of the commercially available Abraxane^®^. When systemically administered in a mouse xenograft model for human breast cancer, the HSA-PEG-based nanoparticle formulation exhibited an extended systemic circulation for more than 96 h and enhanced intratumoral accumulation, resulting in a remarkable anticancer effect and prolonged survival of the animals.

## Introduction

Self-assembled nanostructures have attracted much attention in the field of drug delivery due to their ability to be targeted to the site of disease, thus improving drug pharmacokinetics and enhancing the cellular uptake (Grzelczak et al., 2010). Among the self-assembled structures of various materials, proteins have long been studied and popularly used as building blocks to design self-assembled nanocarriers for small molecules and macromolecules. Protein-based materials are generally regarded as safe for clinical use because of their excellent biocompatibility and biodegradability. In addition, a number of functional groups in the peptide chain of a protein allow for various surface modifications such as covalent or non-covalent attachment of drugs and ligands (Weber et al., 2000; De et al., 2009).

Human serum albumin (HSA, 66.5 kDa), the most abundant serum protein (35–50 g/l) in blood, is a versatile macromolecular carrier for various endogenous compounds with limited solubility, including fatty acids and bilirubin, facilitating their transport throughout the systemic circulation. In addition, the binding of HSA to therapeutic agents, such as taxanes, sulfonamides, penicillin, and benzodiazepines, can greatly affect the biodistribution, bioactivity and metabolism of the drugs (Carter & Ho, 1994; Curry et al., 1998; Neumann et al., 2010). Owing to these features, HSA has been widely employed as a biomaterial with clinically proven safety for designing drug delivery systems (Geny et al., 1993; Sparreboom et al., 2005; Yang et al., 2007; Kratz, 2008). HSA-based nanoparticles can accumulate in solid tumors via the enhanced permeation and retention (EPR) effect and the interaction with 60-kDa glycoprotein (gp60) receptors that are preferentially expressed on tumors (Desai et al., 2006).

Paclitaxel (PTX) is a potent chemotherapeutic agent used as a monotherapy, as well as in a combination therapy regimen for the treatment of a broad range of advanced carcinomas including breast, lung, head and neck, and ovarian cancer (Crown & O'Leary, 2000; Maeda, 2012). Due to its limited water solubility, PTX has been classically formulated with a 50:50 mixture of Cremophor EL (polyoxyethylated castor oil) and ethanol to improve its solubility. However, acute adverse effects such as hypersensitivity reactions and neurotoxicity are associated with the formulation. During the last few decades, a number of formulations including liposomes (Crosasso et al., 2000), polymeric micelles (Zhang et al., 1996; Cho et al., 2004), emulsions (Lundberg 1997), and conjugation with hydrophilic polymer (Fraier et al., 1998) and polypeptides (Yamada et al., 2010) have been introduced. Liposome- and polymeric micelle-based formulations for PTX have been commercialized and in clinical trials (Koudelka & Turanek 2012; Shin et al., 2016). However, conventional liposomes still have drawbacks of complicated quality assurance with high manufacturing cost and rapid *in vivo* clearance by phagocytic reticuloendothelial (RES) systems after systemic administration (Sercombe et al., 2015). Introduction of PEG to liposome surface (PEGylated liposome/PTX) could improve the clearance problem (Uster et al., 1996). Thermodynamic stability of polymeric micelle when the polymer concentration in the blood is lower than its critical micelle concentration (CMC) has still been controversial (Kim et al., 2010; Yokoyama, 2014). Chemical crosslinking and intermolecular hydrogen bonding between polymer chains could be one of the solutions to address the stability issue (Ke et al., 2014). Notably, a Cremophor-free paclitaxel formulation prepared by high-pressure homogenization of the drug with HSA achieved a significant reduction in the risk of adverse effects (Ibrahim et al., 2002; Taylor et al., 2002). The formulation has been approved by the US Food and Drug Administration (FDA) and marketed under the name of Abraxane^®^ for the treatment of metastatic breast cancer and pancreatic cancer.

In this study, PEGylated HSA (HSA-PEG) was used for a facile oil-free fabrication of HSA nanoparticles containing PTX (HSA-PEG/PTX). We hypothesized that the PEGylation of HSA would increase the solubility of the protein in a medium containing water-miscible organic solvents such as tetrahydrofuran, acetonitrile, ethanol, or methanol (Inada et al., 1986) and provide stability to self-assembled nanoparticles containing hydrophobic drugs in a physiological fluid. The HSA-PEG/PTX can be readily self-assembled with a simple film casting and re-hydration process. Due to its improved solubility in various water-miscible solvents or their aqueous mixture, PEGylated HSA can readily dissolve PTX to form a clear thin film after solvent evaporation in which the solvent can be readily recycled. Rehydration of the film resulted in the spontaneous formation of HSA-PEG/PTX without the application of high shear or pressure or additional chemical crosslinking. In addition, the nanoparticles can be stored as a lyophilized powder, which maintains their colloidal stability after reconstitution in a desired injectable medium. The reconstituted nanoparticles demonstrated prolonged systemic circulation following intravenous injection, significant accumulation in the solid tumor region, and remarkable antitumor effects in an animal tumor model. The results suggest that the HSA-PEG-based nanoparticles can be used as a simple, cost-effective, and green theranostic platform with reduced formulation-mediated toxicity for various poorly water-soluble drugs and hydrophobic imaging probes.

## Materials and methods

### Materials

Paclitaxel (PTX) was obtained from Samyang Biopharmaceuticals Co. (Seoul, Korea). Human serum albumin (HSA) and nile red were purchased from Sigma-Aldrich (St. Louis, MO). Methoxy-poly(ethyleneglycol) succimidylglutarate (mPEG-NHS, *M*_W_ 5000) was purchased from SunBio Inc. (Anyang, Korea). Cell culture reagents, including Dulbecco’s modified Eagle’s medium (DMEM), phosphate-buffered saline (PBS, pH 7.4), and trypsin-EDTA, and fluorescent dyes for confocal microscopic imaging, including 4′,6-diamidino-2-phenylindole (DAPI) and 1,1′-dioctadecyl-3,3,3′,3′-tetramethylindocarbocyanine perchlorate (DiIC18), were purchased from Invitrogen (Carlsbad, CA). Abraxane^®^ was purchased from Celgene Co. (Summit, NJ). All organic solvents were of analytical grade and were used without further purification.

### Synthesis of HSA-PEG conjugates

To conjugate PEG to HSA, 75 mg of mPEG-NHS dissolved in PBS was slowly added to a solution containing 100 mg HSA under stirring. The stoichiometric molar ratio (mPEG-NHS: HSA) was 20:1. The reaction was carried out at room temperature for 12 h. The resulting conjugate was dialyzed against deionized water to remove unreacted PEG (MWCO 50,000, Spectrum, Rancho Dominguez, CA), lyophilized, and stored at −20 °C until use. The synthesis of the conjugate was analyzed using SDS-PAGE and gel permeation chromatography (GPC) which was performed using a Waters 626 HPLC pump equipped with a Waters 486 UV detector and PL aquagel OH mixed column (Agilent 1100 s, Agilent, Santa Clara, CA). The mobile phase was phosphate butter saline (PBS) at a flow rate of 1 ml/min.

### Preparation of HSA-PEG/PTX

PTX-loaded HSA-PEG nanoparticles were prepared using a film casting method. HSA-PEG (40 mg) and PTX (4 mg) were dissolved in 10 ml of 50% aqueous tetrahydrofuran (THF) and stirred for 30 min at ambient temperature. The solvent was evaporated to form a film containing HSA-PEG and PTX using a rotary evaporator at 100 °C under reduced pressure. The thin film was dispersed in 20 ml of deionized water by sonication. The resulting solution was filtered through a 0.8 μm cellulose acetate filter unit and lyophilized to obtain homogeneous white powder. HSA-PEG/PTX were then spontaneously generated by dissolving the lyophilized product in PBS. To determine the amount of drug in HSA-PEG/PTX, the nanoparticles dissolved in DMSO were analyzed using a Waters 626 HPLC pump equipped with a Waters 486 UV detector and a LiChrospher 100 RP-18 column (Millipore, Billerica, MA). An acetonitrile − water (45:55, v/v) mixed solvent was used as a mobile phase at a flow rate of 0.5 ml/min. The loading content (%) and loading efficiency (%) was calculated based on the following equations:

$$\text{Loading content }\left(\%\right)=\;\frac{\text{mass of PTX in HSA}-\text{PEG}/\text{PTX}}{\text{total mass of HSA}-\text{PEG}/\text{PTX}}\text{ × }100$$

$$\begin{array}{c} \text{Loading efficiency }\left(\%\right)=\;\\ \frac{\text{actual amount of PTX in HSA}-\text{PEG}/\text{PTX}}{\text{theoretical amount of PTX in HSA}-\text{PEG}/\text{PTX}}\text{ × }100 \end{array}$$

### Characterization of HSA-PEG/PTX nanoparticles

The morphology of HSA-PEG/PTX was observed using both transmission and scanning electron microscopes (TEM and SEM). For TEM analysis, the nanoparticles in deionized water were dropped onto a 300-mesh carbon-coated copper grid and dried at room temperature. The grid was stained with 2% uranyl acetate and observed under a transmission electron microscope (JEM-3010, JEOL, Tokyo, Japan). For SEM analysis, the nanoparticles were placed on a mica surface by spin coating. The sample was coated with gold in the Precision Etching Coating System (Gantan 682 PECS, Gantan, Pleasanton, CA) and analyzed using a field emission scanning electron microscope (JSM-7600F, JEOL, Tokyo, Japan). The hydrodynamic size was determined using a light scattering method (Zeta Plus, Brookhaven Instrument Co., Holtsville, NY) with a He-Ne laser at a wavelength of 632 nm and a 90° detection angle.

### Nile red inclusion assay

Nile red was employed as a polarity-sensitive fluorescent probe to identify the locations of water-insoluble drug in the hydrophobic domains of the HSA-PEG nanoparticles. HSA-PEG/nile red nanoparticles were prepared by the film casting method with the exception of the use of 80% aqueous methanol as a co-solvent. The shift of emission was monitored at room temperature using a spectrofluorometer (RF-5301PC, Shimadzu, Kyoto, Japan) at an excitation wavelength of 550 nm. The spectra were accumulated with an integration of 2 s/nm.

### In vitro release of PTX

The release of PTX from HSA-PEG/PTX was monitored using a dialysis method in the presence of the hydrotropic agent sodium salicylate as a hydrotropic agent (Cho et al., 2004). The freeze-dried HSA-PEG/PTX (10 mg) suspended in 1 ml of deionized water were placed in the dialysis membrane (MWCO 10000, Spectrum, Rancho Dominguez, CA). The drug release was performed in 20 ml of 1.0 M sodium salicylate solution on an orbital shaker at 37 °C. The sample was collected at predetermined time intervals and supplemented with an equal volume of the fresh release medium. The concentration of the drug was determined by HPLC analysis.

### Cell culture and cancer cell proliferation assay

Human breast cancer cells (SK-BR-3, MDA-MB-453, MCF-7) purchased from Korean cell line bank were cultured in DMEM supplemented with 10% FBS and maintained at 37 ^°^C in a humidified 5% CO_2_ atmosphere. The cytotoxic effect of HSA-PEG/PTX on the cancer cells was evaluated by measuring mitochondrial dehydrogenase activity using an MTT assay. The cells were plated in a 96-well plate at a seeding density of 5.0 × 10^3^ cells/well in a growth medium consisting of DMEM with 10% FBS and grown for 24 h at 37 °C. The culture medium was replaced with the fresh growth medium containing 10% FBS. Desired formulations diluted in DMEM were added to the cells. After incubating for 48 h or 72 h, 100 μl of fresh growth medium containing 50 μg MTT were substituted to each well, and the cells were incubated at 37 °C for 2 h. The insoluble formazan crystals were dissolved in DMSO. Absorbance was measured at 490 nm in a microplate reader (EL 808, Bio-Tek Instrument, Winooski, VT). Survival percentage was calculated in comparison to the mock-treated cells (100% survival).

### Confocal microscopy

To visualize the cellular uptake of HSA-PEG nanoparticles, an alkylated fluorescence probe, DiIC_18_, was used as a hydrophobic imaging probe. HSA-PEG/DiIC_18_ was also prepared by a film casting method. HSA-PEG (40 mg) and DiIC_18_ (4 mg) were mixed in 10 ml of 50% aqueous tetrahydrofuran (THF), followed by stirring for 30 min. After solvent evaporation, the thin film containing HSA-PEG and DiIC_18_ was dispersed in deionized water by using a sonifier. The solution was purified using a cellulose acetate filter and subsequently lyophilized. For formation of HSA-PEG/DiIC_18_ nanoparticle, the lyophilized resultant was dissolved in PBS. HSA-PEG/DiIC_18_ nanoparticles (100 μg/ml) were treated to MCF7 cells seeded in confocal imaging dishes (35 mm Glass Bottom Dish; SPL Life Sciences, Gyeonggi-do, Korea) at a density of 2.5 × 10^4^ cells/well and grown in DMEM supplemented with 10% FBS at 37 °C. After 4 h incubation, the cells were washed three times with PBS. The nuclei were stained with DAPI (4',6-diamidino-2-phenylindole, Invitrogen, Carlsbad, CA). The cells were then fixed in 10% buffered formaldehyde (Hedwin Corp., Baltimore, MD) at 4 °C for 30 min and washed three times with cold PBS. The subcellular localization of the nanoparticles was visualized by laser scanning confocal fluorescence microscopy using a Zeiss LSM 510 microscope (Carl Zeiss MicroImaging GmbH, Jena, Germany). An oil immersion objective lens was used for the epidetection configuration.

### Animal experiments

To evaluate the therapeutic effect of HSA-PEG/PTX, female nude mice (BALB/c nu/nu) were purchased from Japan SLC (Hamamatsu, Japan) and used at 7 weeks of age. All animal experiments were performed in accordance with the guidelines provided by the Institutional Animal Care and Use Committee at Sungkyunkwan University. To generate a tumor xenograft animal model, freshly harvested SK-BR-3 cells (3 × 10^6^ cells per mouse) were subcutaneously inoculated into the haunch of the mice. When a tumor size of ∼100 mm^3^ was attained, HSA-PEG/PTX and the control formulations (PTX and Abraxane^®^) were administered via a tail vein on days 0, 3, 5, 7, and 9. For PTX treatment as a control, 5 mg of PTX was dissolved in 100 μl DMSO and subsequently added to 900 μl deionized water. The dose amount of PTX was 4 mg/kg body weight for each formulation. The tumor growth was monitored up to 35 d by measuring perpendicular diameters using a caliper. The tumor volume was calculated using the following equation: tumor volume (mm^3^) = [length × (width)^2^]/2.

To observe *in vivo* biodistribution and tumor accumulation of the nanoparticles, a lipophilic fluorescent probe, DiIC_18_, was used as a probe for *in vivo* optical imaging (HSA-PEG/DiIC_18_). The HSA-PEG/DiIC_18_ was injected into mice bearing a SK-BR-3 tumor via the lateral tail vein. At predetermined time intervals, *in situ* fluorescence images of each mouse were obtained using the Optix MX3 system (Advance Research Technologies, Montreal, QC). The images were analyzed using the OptiView^®^ software (Optiview, Inc., Jacksonville, FL).

## Results and discussion

**Figure 1.**
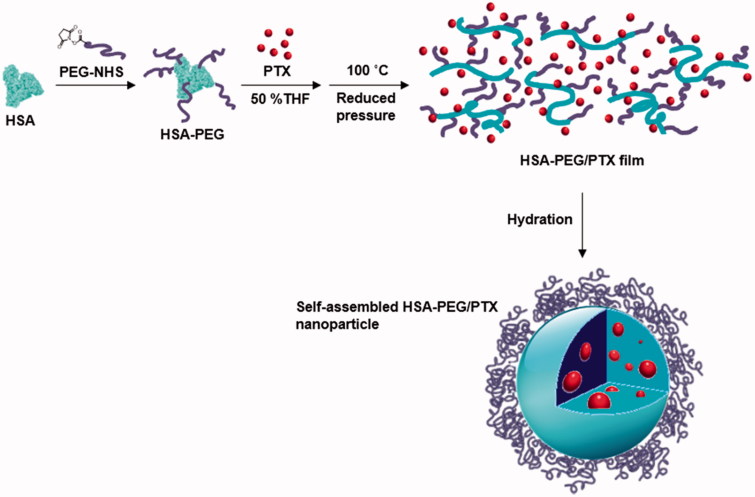
Schematic illustration of the formation of HSA-PEG/PTX nanoparticles.

**Figure 2.**
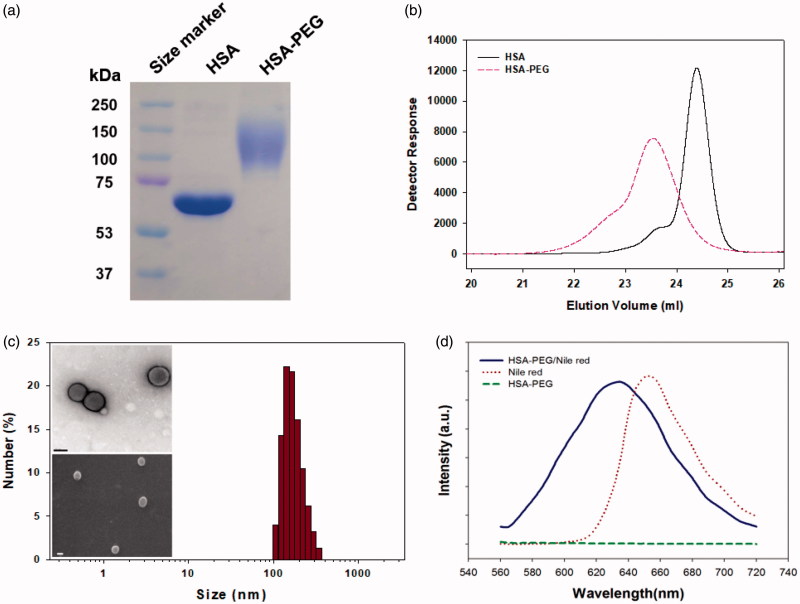
Characterization of the HSA-PEG conjugate. (a) SDS-PAGE. (b) HPLC analysis. (c) Size distribution and morphology of HSA-PEG/PTX determined by the light scattering method and electron microscopy (upper insets: TEM image; lower inset: SEM image). (d) Nile red inclusion assay for the observation of the localization of the hydrophobic model dye, nile red, in HSA-PEG/PTX.

The preparation for HSA-PEG nanoparticles encapsulating PTX is illustrated in Figure 1. HSA-PEG was synthesized in an aqueous buffer solution by conjugating NHS-derivatized methoxy PEG (mPEG-NHS, *M*_W_ 5000) to the primary amine groups of HSA. The molecular weight of HSA-PEG ranged from approximately 100 to 150 kDa, which corresponds to 7 to 16 PEGs conjugated to one HSA molecule, as determined by SDS-PAGE and GPC analysis (Figure 2(a,b)). The improved solubility of PEGylated HSA in various organic solvents and their aqueous mixtures was reported in previous literature (Inada et al., 1986). To generate HSA-PEG/PTX nanoparticles, HSA-PEG conjugate and PTX dissolved in 50% tetrahydrofuran (THF) aqueous solution were subjected to a rotary evaporator for the evaporation of THF and water at an elevated temperature (100 °C), forming a clear and transparent film without any visible sign of precipitation or granulation. It should be noted that the evaporated organic solvent can be readily collected and reused. The dissolution of HSA-PEG in 50% THF may cause the denaturation of the HSA chain, leading to the exposure of the hydrophobic moieties. The HSA-PEG-PTX were generated by localization of PTX in the hydrophobic moieties of HSA-PEG dissolution during the solvent evaporation and subsequent film formation. The PTX molecules would be localized in the hydrophobic moieties during the solvent evaporation and subsequent film formation (Figure S1(A) see Supplementary information). The film can be readily dissolved to form a transparent nanoparticle solution in deionized water. The nanoparticles can also be stored as a lyophilized form, which can be readily reconstructed to a clear aqueous solution containing nanoparticles by adding PBS or physiological saline (Figure S1(b), the dissolution movie of HSA-PEG/PTX is available in Supplementary data). The size and the surface charge of resulting HSA-PEG/PTX were 176 ± 3.6 nm (Figure 2(c)) and −2.57 ± 0.31 mV, respectively, as determined by a light scattering method. The spherical morphology of the nanoparticle was observed by transmission and scanning electron microscopy (insets in Figure 2(c)).

The localization of PTX in the hydrophobic domains of HSA-PEG was simulated using a hydrophobic fluorescent probe, nile red, of which the fluorescence spectrum is highly influenced by its microenvironment (Sackett & Wolff, 1987). The blue shift of the emission spectrum of nile red indicates that the fluorescent probe is surrounded by the hydrophobic amino acids residues of HSA (Figure 2(d)). In the same way, it can be presumed that PTX can also be localized at the hydrophobic domains of HSA-PEG, which may effectively prevent the formation and growth of PTX crystals in the film. The surface of the nanoparticles would be stabilized by the PEG segments forming the outermost hydrophilic layer. According to HPLC analysis, the loading amount and efficiency of nanoparticle were 8.7 wt% and 86.6%, respectively.

**Figure 3.**
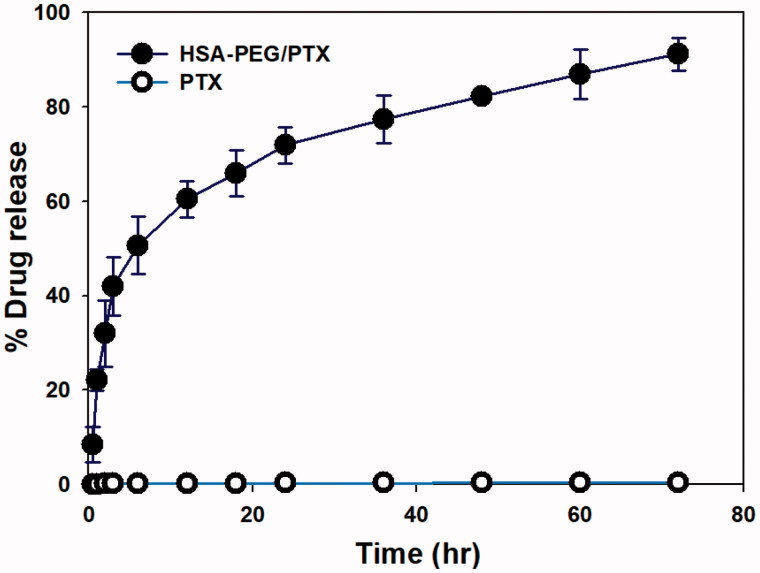
Cumulative release of PTX from HSA-PEG/PTX and dissolution of PTX powder suspension in an aqueous solution. The release experiment was carried out in the presence of 1.0 M sodium salicylate as a hydrotropic agent.

The release of PTX from the HSA-PEG/PTX was observed using a dialysis method in the presence of 1.0 M sodium salicylate, a hydrotropic agent that enhances aqueous solubility of PTX without compromising the structural property of the self-assembled micelles (Lee et al., 2003; Cho et al., 2004). The sodium salicylate was used to maintain a sink condition for PTX released from HSA-PEG/PTX (Cho et al., 2004). While PTX was released from HSA-PEG/PTX in a sustained fashion, only a limited amount of the drug was dissolved from PTX power suspended in the release medium, suggesting that the increased solubility of the poorly water soluble drug was attributed to the increased surface area of HSA-PEG/PTX due to its nano-scale dimension (Figure 3).

**Figure 4.**
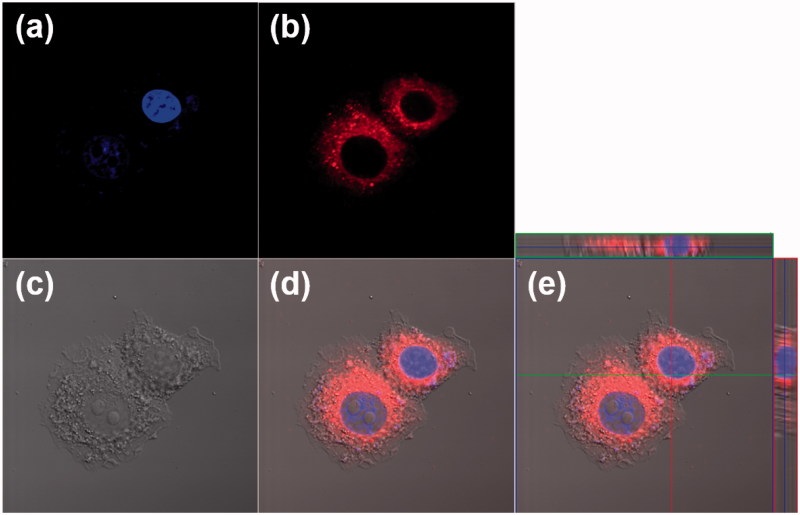
Cellular uptake of HSA-PEG/DiIC_18_ in SK-BR-3cells. The images were obtained by a confocal laser scanning microscope 4 h after the treatment of HSA-PEG/DiIC_18_. (a) DAPI (blue), (b) DiIC_18_ (red), (c) differential interference contrast (DIC) image, (d) merged image, and (e) merged image with orthogonal z-stack projection.

The cellular uptake of HSA-PEG nanoparticles was visualized by confocal microscopy using a lipophilic fluorescent probe (DiIC_18_) as a hydrophobic model drug incorporated in HSA-PEG nanoparticles (HSA-PEG/DiIC_18_). Owing to its hydrophobicity, DiIC_18_ is often used to stain cell membranes. HSA-PEG/DiIC_18_ can be readily taken up by human breast cancer cells (SK-BR-3) (Figure 4). It should be noted that the fluorescence is mostly localized to the cytosolic space after a 4 h incubation rather than the cell plasma membrane, suggesting that HSA-PEG nanoparticle may be delivered into the cells without losing integrity. If the nanoparticles were dissociated and, therefore, released the fluorescent probe before being taken up by the cells, the fluorescent signals would also have been observed in both cell membrane and cytosol.

**Figure 5.**
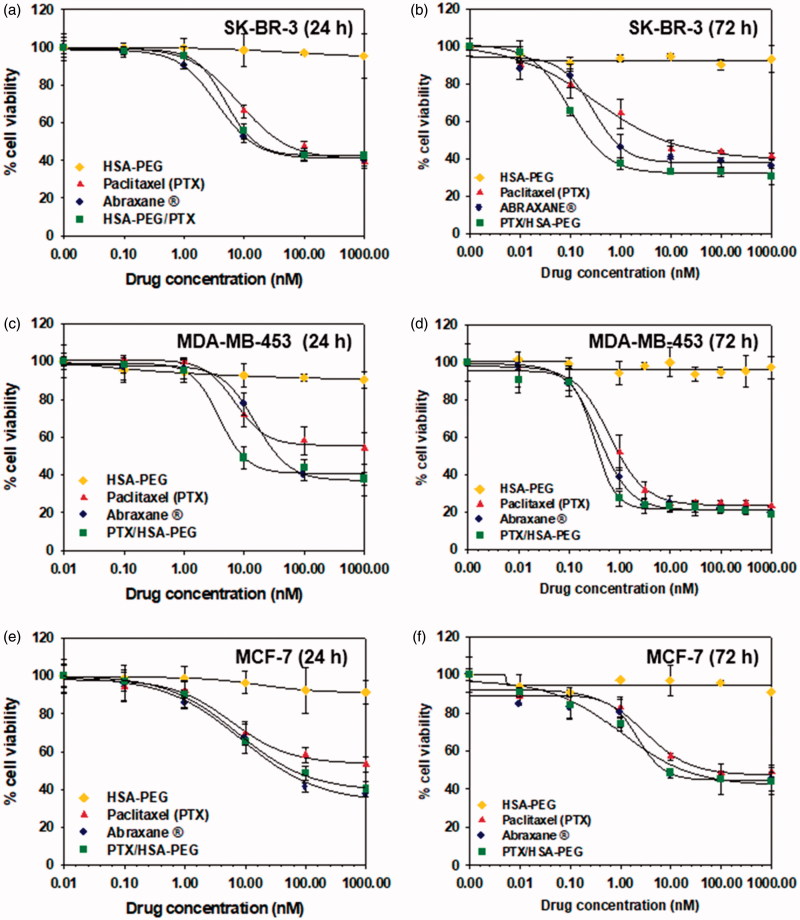
Cytotoxicity of HSA-PEG/PTX in various human breast cancer cells. Cell viability was determined by MTT assays after 24 h (a–c) and 72 h (d–f) incubation with the indicated formulation. (a, d) Cytotoxicity of HSA-PEG/PTX in SK-BR-3 cells. (b, e) Cytotoxicity of HSA-PEG/PTX in MDA-MB-453 cells. (c,f) Cytotoxicity of HSA-PEG/PTX in MCF-7 cells. HSA-PEG, PTX dissolved in DMSO and Abraxane^®^ were used as controls.

The cytotoxic activity of HSA-PEG/PTX was evaluated in three different human breast cancer cell lines (SK-BR-3, MDA-MB-453, and MCF-7) and compared with that of HSA-PEG conjugate, commercially available Abraxane^®^, and free PTX dissolved in DMSO. HSA-PEG/PTX nanoparticle demonstrated comparable or even higher cytotoxicity in each cell line compared with Abraxane^®^ (Figure 5). The IC_50_ values (72 h) of PTX dissolved in DMSO, Abraxane^®^, and HSA-PEG/PTX in SK-BR-3 cells were 7.36 nM, 0.72 nM, and 0.23 nM, respectively (Figure 5(d)). The carrier (HSA-PEG) did not show any detectable cytotoxicity in all cell lines.

**Figure 6.**
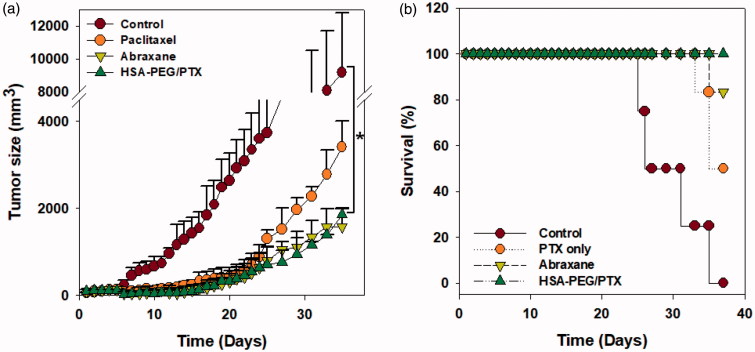
Inhibition of tumor growth by HSA-PEG/PTX in an animal tumor model. (a) Inhibition of SK-BR-3 tumor growth by HSA-PEG/PTX. One hundred microliters of the desired formulations were systemically administered to tumor-bearing mice on days 0, 3, 5, 7, and 9. Tumor growth was monitored by measuring tumor volume as described in the Materials and methods section and expressed as a percent of the initial volume (100 mm^2^). The dose of PTX was 4** **mg/kg. The results are presented as mean ± SD (*n*** **=** **8/group). *p < .01 versus controls. (b) Mouse survival rate after the systemic administration of PTX, Abraxane^®^, and HSA-PEG/PTX.

The *in vivo* anticancer effect of HSA-PEG/PTX nanoparticles was further evaluated in a mouse model bearing a SK-BR-3 tumor xenograft after intravenous injection through tail vein. Each formulation was systemically administered five times to the tumor-bearing mice in the early stage of tumor growth (days 0, 3, 5, 7, and 9). The administered dose was 4 mg PTX/kg. In accordance with the results from *in vitro* study, both HSA-PEG/PTX nanoparticles and Abraxane^®^ effectively inhibited solid tumor growth (Figure 6(a)). Survival rate of the tumor-bearing animals was also significantly improved in HSA-PEG/PTX nanoparticle- and Abraxane^®^-treated groups (Figure 6(b)). No death was observed during the experimental period in group of mice treated with HSA-PEG/PTX nanoparticles.

**Figure 7.**
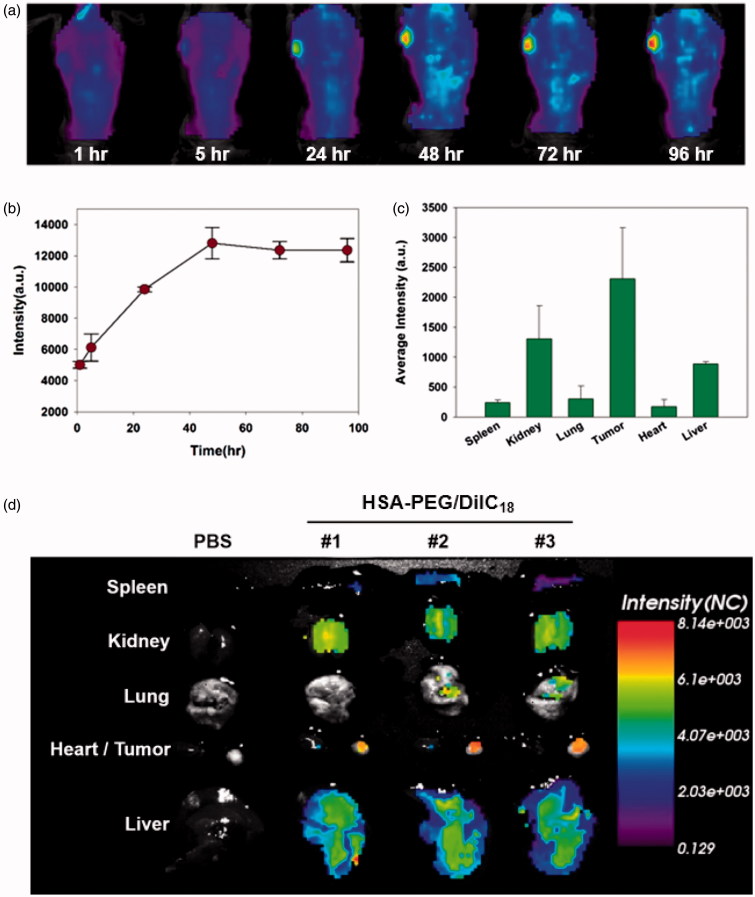
*In vivo* biodistribution of HSA-PEG/DiIC_18_ nanoparticles. (a) Extended systemic circulation and tumor accumulation of HSA-PEG/DiIC_18_ after IV injection through the tail vein. The image was obtained by *in situ* whole body fluorescence imaging, and the tumor accumulation profile was analyzed using OptiView^®^ software (b). (c) *In vivo* biodistribution of HSA-PEG/DiIC_18_ nanoparticles in the major organs. The average fluorescence intensity of each organ was obtained from *ex vivo* biodistribution images shown in (d) and represented as mean ± SD (*n*** **=** **3).

To observe the *in vivo* biodistribution profiles of HSA-PEG-based nanoparticles, DiIC_18_-incorporated HSA-PEG nanoparticles (HSA-PEG/DiIC_18_) were prepared and intravenously administered through tail veins of SK-BR-3 tumor-bearing mice. The nanoparticles were accumulated in the tumor for up to 96 h, suggesting circulation of the nanoparticles for an extended period of time (Figure 7(a,b)), and the highly flexible hydrophilic layer of the PEG effectively protected the protein core from recognition and clearance by reticuloendothelial systems (RES). Multiple inter-and intra-peptide chain hydrogen bonds may also contribute the stability of the self-assembled nanostructure during the systemic circulation. The incorporation of hydrogen bonding functionalities in the hydrophobic core of amphiphilic block copolymers resulted in the stabilized micelles with reduced CMC values, suggesting the significant role of non-covalent interactions in the stability of supramolecular nano-assemblies for systemic drug delivery (Kim et al., 2010; Yang et al., 2010). Figure 7(c) shows *ex vivo* evaluation of the HSA-PEG/DiIC_18_ nanoparticle distribution in major organs including the tumor, heart, liver, lungs, spleen, and kidneys at 48 h after systemic administration. The fluorescence signals were mostly detected in the tumor, liver, and spleen. The near-infrared *in vivo* fluorescence imaging also demonstrated the biodistribution of HSA-PEG/DiIC_18_ in the tumor-bearing mice (Figure 7(d)). As compared with the color histogram, the strongest fluorescence signals were observed in the tumor, although significant fluorescence intensity also appeared in the liver and kidneys. A relatively large amount of HSA-PEG/DiIC_18_ was localized in the tumor site, possibly attributed to the enhanced permeation and retention (EPR) effect due to the leaky vasculature in the vicinity of solid tumor (Matsumura & Maeda, 1986). The safety and biocompatibility of the carrier material, HSA-PEG, was further assessed by administering excess amount of HSA-PEG into mice through the tail vein (500 mg/kg). HSA-PEG, did not elicit significant toxicities in major organs, including the liver, spleen, heart, lungs, and kidneys, after intravenous administration (Figure S2 see Supplementary information). These results suggest that the HSA-PEG-based nanoparticle can be considered as a safe and long-circulating carrier for poorly water-soluble drugs.

## Conclusions

HSA-PEG/PTX nanoparticles were readily fabricated by a simple film casting and re-hydration method, which was used for oil-free manufacture to avoid the adverse hypersensitivity and dose-limiting toxicities associated with solvent-based formulations. The formulation can be stored as a lyophilized powder and reconstituted in an aqueous buffer prior to use. The HSA-PEG/PTX was able to mediate efficient cellular uptake and demonstrated a comparable or better cytotoxic effect in various cancer cells compared with the commercially available formulation, Abraxane^®^. Moreover, the HSA-PEG/PTX formulation achieved a prolonged systemic circulation after intravenous administration and significant intratumoural accumulation in a tumor-bearing mouse model, leading to a desirable anticancer effect and improved survival of the animals. Thus, the current research provides an alternative theranostic platform for solid tumor imaging and effective cancer therapy using PTX with minimal adverse effects for stable chemotherapy. In addition, since PTX can be considered a representative drug with limited water solubility, the currently suggested formulation could also be used as a potent platform for the dissolution of various poorly water-soluble drugs in aqueous media and the improvement of *in vivo* pharmacokinetic behavior of the drugs.

## Supplementary Material

Supplementary data

Supplementary data
