# Supplementary material for: Self-assembled PEGylated albumin nanoparticles (SPAN) as a platform for cancer chemotherapy and imaging
Source: Drug Deliv. 2018 Jul 25;25(1):1570–8. doi: 10.1080/10717544.2018.1489430 (PMC6060380; doi:10.1080/10717544.2018.1489430)
Supplement: Supplementary data [file IDRD_A_1489430_SM5146.docx]

Supplementary data

**Self-assembled PEGylated albumin nanoparticles (SPAN) as a platform for cancer chemotherapy and imaging**

Jung Eun Leea, Myung Goo Kim, Yeon Lim Jang, Min Sang Lee, Nak Won Kim, Yue Yin, Jong Han Lee, Su Yeon Lim, Ji Won Park, Jaeyun Kim, Doo Sung Lee, Sun Hwa Kim, Ji Hoon Jeong


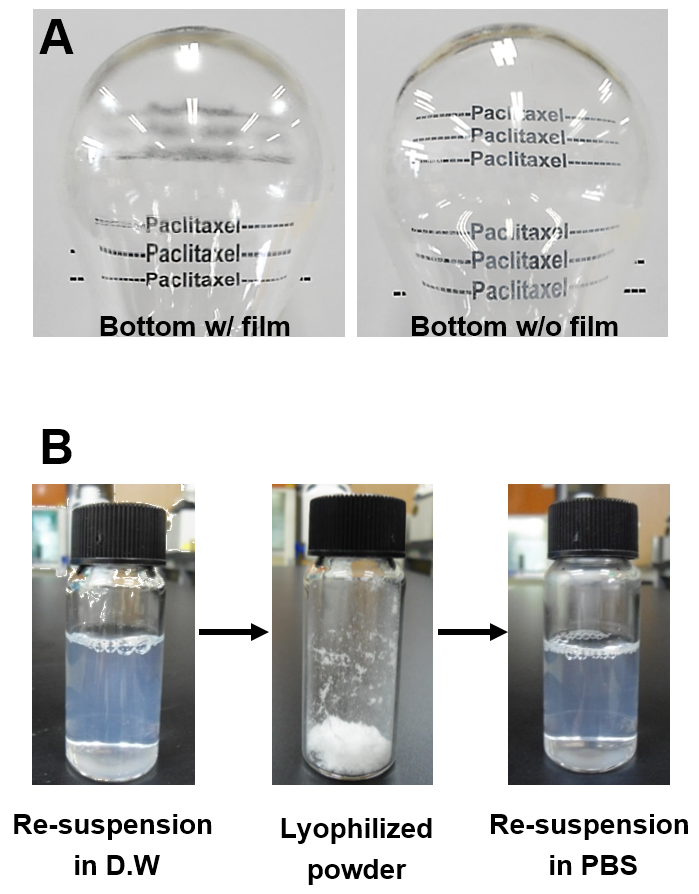


**Fig. S1.** Preparation of HSA-PEG/PTX nanoparticles by film casting and re-hydration method. (A) Formation of HSA-PEG/PTX film in the round-bottomed flask by solvent evaporation. (B) Lyophilized form of the HSA-PEG/PTX nanoparticles (middle) and reconstruction of the nanoparticles in PBS from the lyophilized powder (right).


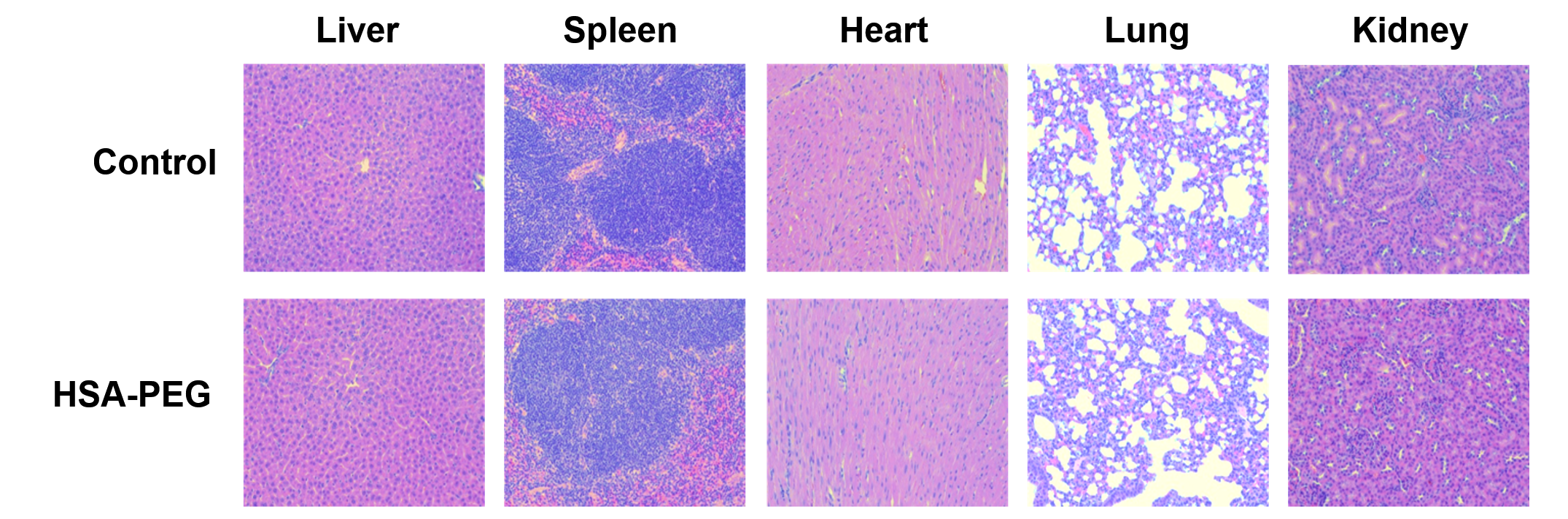


**Fig. S2.** Histological evaluation (H&E staining) of paraffin sections of the liver, spleen, heart, lungs and kidneys 24 h after intravenous injection of HSA-PEG (500 mg/kg).
